# Neurotransmitters crosstalk and regulation in the reward circuit of subjects with behavioral addiction

**DOI:** 10.3389/fpsyt.2024.1439727

**Published:** 2025-01-14

**Authors:** Zhenlei Peng, Qiyu Jia, Junxiong Mao, Xiao Luo, Anqi Huang, Hao Zheng, Shijie Jiang, Qi Ma, Chuang Ma, Qizhong Yi

**Affiliations:** ^1^ Xinjiang Clinical Medical Research Center of Mental Health, The Psychological Medicine Center, The First Affiliated Hospital of Xinjiang Medical University, Urumqi, Xinjiang, China; ^2^ Department of Trauma Orthopedics, The First Affiliated Hospital of Xinjiang Medical University, Urumqi, Xinjiang, China; ^3^ Child Mental Health Research Center, Nanjing Brain Hospital, Clinical Teaching Hospital of Medical School, Nanjing University, Nanjing, China; ^4^ The First Affiliated Hospital of Zhejiang Chinese Medical University (Zhejiang Provincial Hospital of Chinese Medicine), Zhejiang, China; ^5^ Xinjiang Key Laboratory of Metabolic Disease, Clinical Medical Research Institute, The First Affiliated Hospital of Xinjiang Medical University, Urumqi, China

**Keywords:** behavioral addictive disorders, reward circuit, neurotransmitters, dopamine, crosstalk

## Abstract

Behavioral addictive disorders (BADs) have become a significant societal challenge over time. The central feature of BADs is the loss of control over engaging in and continuing behaviors, even when facing negative consequences. The neurobiological underpinnings of BADs primarily involve impairments in the reward circuitry, encompassing the ventral tegmental area, nucleus accumbens in the ventral striatum, and prefrontal cortex. These brain regions form networks that communicate through neurotransmitter signaling, leading to neurobiological changes in individuals with behavioral addictions. While dopamine has long been associated with the reward process, recent research highlights the role of other key neurotransmitters like serotonin, glutamate, and endorphins in BADs’ development. These neurotransmitters interact within the reward circuitry, creating potential targets for therapeutic intervention. This improved understanding of neurotransmitter systems provides a foundation for developing targeted treatments and helps clinicians select personalized therapeutic approaches.

## 1 Introduction

With social progress and technological development, significant shifts have occurred in people’s lifestyles and behavior patterns. Concurrently, the prevalence of behavioral addictive disorders (BADs) is increasing, presenting with a wide range of manifestations. This trend is particularly noticeable among younger demographics, where studies indicate that approximately 1.25% to 5% of minors meet the criteria for Internet Gaming Disorder (IGD) (1). In the 11th Revision of *the International Classification of Diseases* (ICD-11) (2) issued by the World Health Organization (WHO), gambling disorder (GD) and IGD have been classified as disorders caused by addictive behaviors, sparking widespread research interest. Research indicates that other non-substance addictive behaviors, such as compulsive sexual behavior disorder (CSBD) (3), buying-shopping disorder (4) and internet-use disorders (5), show similar clinical presentations and neurobiological mechanisms to gambling and gaming addictions. The defining feature of BADs is the repetitive engagement in rewarding behaviors, accompanied by weakened control despite negative consequences. Moreover, BADs may share clinical, genetic, neurobiological, and phenomenological similarities with substance addictions. Recent meta-analyses have revealed that (6) both conditions exhibit disrupted resting-state functional connectivity between the frontal network and other high-level cognitive networks (including default mode, affective, and salience networks). Therefore, studying the neurobiological basis of BADs and exploring their mechanisms and treatment approaches has significant theoretical and practical implications similar to substance addictions.

BADs and substance addictions share a common core feature: the loss of behavioral control (7). This significant overlap in their manifestations implies a shared neurobiological basis involving the disruption of the “reward system,” also known as the reward circuit. In contrast to previous perspectives, it is now acknowledged (8) that individuals suffering from addiction are not addicted to a specific substance or activity but rather to the corresponding brain responses elicited. The reward circuit encompasses an intricate network of neurons governing human reward and punishment responses through interconnected pathways. It encompasses multiple brain regions, including the ventral tegmental area (VTA), nucleus accumbens (NAc)in the ventral striatum (VS), basal ganglia, prefrontal cortex (PFC), amygdala (AMY), and hippocampus. Among these regions, the mesolimbic dopamine pathway in the midbrain serves as the ultimate common pathway for reinforcement and reward triggered by physiological stimulation or addictive behaviors (9). In the development and maintenance of BADs, specific brain regions establish connectivity via neurotransmitter-mediated signaling, resulting in intricate individualized neurobiological changes.

In the past few decades, dopamine (DA) has held a prominent position as the central factor in BADs, playing a pivotal role within the reward circuit. Nevertheless, recent studies have underscored the significance of other neurotransmitters and their intricate interplay within the reward circuit, including serotonin (i.e., 5-hydroxytryptamine, 5-HT), endorphins, gamma-aminobutyric acid (GABA), glutamate (Glu), and norepinephrine (NE) (10, 11). The coordinated interactions among these neurotransmitters, particularly the DA-glutamate-GABA circuit in the NAc and the serotonin-DA interactions in the VTA, form the neurochemical basis of the reward circuit (12, 13). Furthermore, the interactions between neurotransmitters can also affect the activity of the reward circuit and the development and maintenance of BADs through mechanisms such as synaptic plasticity, neuronal excitability, membrane potential, and receptor binding (12, 13). Recent neuroimaging and molecular studies (6, 14, 15) have revealed specific neural pathways in BADs’ reward circuit, establishing direct links between psychological symptoms and their neurobiological underpinnings.

This review explores the roles of neurotransmitters such as DA, serotonin, endorphins, GABA, Glu, and NE in the reward circuit, focusing on elucidating the intricate interactions between these neurotransmitters. Additionally, this study will analyze the impact of neurotransmitter interactions on reward circuitry activity and BADs, providing insights and references for the neurobiological research of BADs and theoretical support for developing novel treatment strategies targeting specific neurotransmitter interactions.

## 2 Neurotransmitter interactions in BADs

### 2.1 Dopamine

The neurobiological mechanisms associated with the reward circuit in BADs remain partially understood. DA has been identified as a pivotal factor in reward processing, motivation control, and behavioral activation in BADs (16). The dopaminergic system (17, 18) includes the mesolimbic system (from the VTA to the NAc, AMY, and hippocampus), the mesocortical system (from the VTA to the PFC), the nigrostriatal system (from the substantia nigra pars compacta (SNc) to the striatum), and the tuberoinfundibular system, with the mesolimbic system playing a major role in the reward circuit. The VTA, where dopaminergic neurons are located, informs the organism whether environmental stimuli (such as natural rewards, substance abuse, online gaming, stress, etc.) are aversive or beneficial. Nerve propagation takes place from the VTA which leads to DA release in the NAc, and this dopaminergic signaling contributes to feelings of pleasure and contentment. These sentiments enhance motivation to pursue rewards. Current literature indicates (19–22) that individuals with gambling addiction may display inaccurate reward predictions or heightened uncertainty concerning rewards, leading to phasic, peak DA signals within the NAc. Moreover, prolonged and excessive engagement with the internet and video games can swiftly elevate DA release in the NAc. The PFC, encompassing the dorsolateral PFC and orbitofrontal cortex, is another critical brain region linked to BADs. Following DA release in the NAc by VTA neurons, the PFC contributes to decision-making and emotional regulation. For instance, it may construe addictive behaviors as manifestations of pleasure (reward) (20). Simultaneously, exposure to visually stimulating reward cues or pleasurable experiences during gaming triggers an upsurge in DA release within the VS (23–25). In summary, with prolonged exposure to specific activities or stimuli, brain regions integral to the reward circuit interpret signals and provide responses. The pathological surge in DA signaling within regions like the PFC and VS areas constitutes one of the underlying mechanisms of BADs (26).

Elevated DA transmission is crucial in the pathology of BADs. Research findings (27–29) are converging despite lacking cellular-level consensus. In pathological gambling (30–34), the intensity of gambling symptoms shows a positive correlation with heightened DA release within the VS and dorsal striatum (DS). Linnet et al. (35) identified a direct correlation between DA release and subjective excitement. In GD (36), D1 receptor activation in the direct striatal pathway and D2 receptor inhibition in the indirect pathway increase NAc dopamine, affecting decision-making. Moreover, the binding of DA to inhibitory receptors D2 and D3 in the NAc or PFC is closely associated with the impulsive characteristics of GD (37, 38). In addition to binding with receptors in the NAc, DA has been found to increase the severity and impulsivity of GD when it binds with D3 receptors in the SNc (34, 39). Experiments in animal models have also revealed a relationship between D4 receptors and impulse control and gambling behavior (40–42). Furthermore, a study showed (43) that the dopamine transporter (DAT) plays a role in gambling addiction, as the impulsivity of elderly individuals with GD is positively correlated with DAT activity. The clinical manifestations of GD are complex and diverse. It is not only associated with increased synthesis and release capacity of DA in the reward circuitry but also closely linked to the abundance of presynaptic DAT and the availability of postsynaptic receptors. Studies (44–48) of IGD show similar patterns, with increased DA secretion relating to impulsivity and reward dependence. Additionally, both the density and availability of DA receptors in the striatum are decreased in IGD patients compared to healthy controls. It has also been found that longer durations of internet gaming addiction can lead to a more severe imbalance of D2 receptors. Furthermore, the severity of internet gaming addiction and depressive mood negatively correlate with DAT levels (49, 50). In summary, the prolonged use of internet gaming can dysregulate the DA system, exacerbating the severity of IGD and increasing susceptibility to impulsive behaviors.

In contrast, reduced release of DA induces cravings for DA, increasing their vulnerability to engage in impulsive and compulsive behaviors. Resulting in imbalanced decision-making, triggering subsequent releases of additional DA. For example, a study using positron emission tomography (PET) with the tracer [11C] raclopride (51) found that in healthy control subjects, there was a positive correlation between DA release in the VS and performance on the Iowa Gambling Task (IGT) while individuals with GD exhibited an inverse correlation. This suggests that diminished DA release contribute to the imbalanced decision-making observed in GD individuals within rewarding scenarios. Other studies (52, 53) have supported this notion by demonstrating that reduced DA synthesis and release are associated not only with imbalanced decision-making but also with heightened craving and dependence in patients. The intensified craving seen in individuals with BADs is not only linked to reduced DA release but also to a decrease in the density of D2 receptors (54). Reduced binding of D2 and D3 receptors within the VS can lead to heightened craving in gambling addiction patients, accompanied by the emergence of negative emotions such as anxiety and depression. The availability of DA receptors is inversely correlated with emotional impulsivity (“urgency”) in the striatum (30, 37, 55). Moreover, prolonged internet use or video gaming (20, 45) can lead to a decline in DA receptor sensitivity and frontal lobe dysfunction, resulting in cravings for games and triggering negative emotions in individuals with internet gaming addictions. Studies of CSBD neurobiology, including dopaminergic pathways, represent an emerging field, requiring further investigation to establish specific mechanisms (56). Although some studies indicate no significant differences in DA release within the reward circuitry (34, 37, 38) and the availability of D2 and D3 receptors (32, 33) when compared to healthy individuals, the pivotal role of the DA system in BADs remains indisputable based on the existing literature.

In summary, BADs emergence involves DA synthesis, release, receptor availability, DAT, and enzymatic function. DA system disruption and heightened reward circuit activity underlie control loss and impulsivity in BADs. The DA system serves as both the neurobiological foundation of BADs and a significant target for their treatment. This establishes a theoretical framework for the utilization of pharmacological interventions in BADs from a neurobiological standpoint in clinical settings. Specifically, D2 receptors play a role in the interaction with DA release within the reward circuitry of BADs. Several studies have indicated that the use of dopaminergic drugs (such as levodopa) in the treatment of Parkinson’s disease and restless leg syndrome may increase the likelihood of developing gambling problems or other potential impulsive or compulsive behaviors, such as shopping, sex, binge eating, etc. (57–61). Symptom severity in BADs patients also tends to decrease when dopaminergic medications are reduced. Furthermore, the heightened risk of BADs associated with levodopa is not solely attributed to its binding with D2 receptors but also to its inhibition of GABA release, a critical element (62). Some conflicting evidence exists regarding the effects of dopamine D2 receptor antagonists (such as haloperidol, olanzapine, etc.). While one study found (63) that haloperidol reduced the inclination of individuals with GD to place more aggressive bets after receiving a reward in a slot machine task, another study reported (64) that haloperidol heightened the rewarding effects and gambling desires reported by individuals with GD. Additionally, olanzapine has not demonstrated positive effects in the treatment of GD (65–67). These findings highlight the intricate connection between dopamine D2 receptor function and gambling-related motivations and behaviors. Another subset of DA receptors, the D3 receptors (68), are highly concentrated in regions like the NAc, olfactory bulb, and hypothalamus (HYP) and are involved in functions such as reward processing, craving, and aversive emotions. DA agonists like pramipexole and ropinirole, which selectively target D3 receptors, increase the susceptibility to developing GD (39, 69–72). An experimental study using the rat gambling task (rGT) found that buspirone (a 5-HT1A receptor agonist) at a low dose level (3mg/kg) exclusively occupied D3 receptors, while at a high dose level (10mg/kg) simultaneously occupied D2 and D3 receptors, resulting in a greater number of advantageous responses than disadvantageous responses in the rGT for rats (73). In addition, the D4 receptors are also associated with gambling behavior (74). Activation of D4 receptors by the D4 agonist PD168077, primarily in the anterior cingulate cortex (ACC), led to rats displaying incorrect reward expectations during a rodent slot machine task. Since D4 receptor activation promotes gambling-like behaviors, this discovery suggests that D4 receptor antagonists might be promising therapeutic agents for treating BADs. DAT is a crucial protein that regulates DA levels in the synaptic cleft and controls the duration of DA signaling (75). In normal conditions, DAT reuptakes DA from the synaptic gap into the cytoplasm of presynaptic neurons, and alterations in DAT function significantly influence both intracellular and extracellular DA concentrations. Amphetamine is a dopaminergic drug (76) that not only affects synaptic plasticity in dopaminergic neurons but also causes reversal of the direction of DAT and dopamine release in the striatum, which can increase the concentration of DA in the synaptic cleft and may promote adverse effects such as gambling thoughts and behaviors. Another central nervous system stimulant, methylphenidate, inhibits the degradation enzymes of both DA and NE, extending their presence in the synaptic cleft and augmenting their effects. An 8-week trial administering methylphenidate to children diagnosed with attention deficit hyperactivity disorder (ADHD) and internet gaming addiction demonstrated significant reductions in both internet addiction scores and usage time (77), suggesting a potential for methylphenidate to be utilized as a beneficial intervention for internet addiction in children with ADHD. Additionally, supplementation therapy addressing compromised DA function has shown promise in fostering sustained dopaminergic activation, effectively treating impulsive behaviors associated with BADs without adverse effects (78). In the clinical treatment of BADs, attention should also be given to D1 and D5 receptors and the catechol-O-methyltransferase (COMT) enzyme. However, it is important to interpret the aforementioned findings cautiously, as further experimental investigations are required to comprehensively comprehend the role of DA in various BADs to identify more precise targets for prevention and treatment.

Current addiction theories maintain some unresolved viewpoints regarding DA’s role. Beyond DA research, exploring other neurotransmitters is crucial for understanding BADs reward circuitry. A previous study indicated (79) that systems involving serotonin, endorphins, Glu and GABA are associated with substance addictions to varying degrees. Furthermore, these systems (11) exert significant interference on BADs and may interact with DA and other neurotransmitter systems in complex ways.

### 2.2 Serotonin (5-HT)

In addition to the DA system, there is compelling evidence (80) implicating the serotonergic system (the 5-HT system), in the reward circuitry of BADs, playing a role in the initiation and cessation of addictive behaviors. The dorsal and median raphe nuclei (DRN/MRN), located in the brainstem, are the primary sources of serotonergic neurons. These neurons project widely throughout the brain, particularly to key reward-related regions including the NAc, and AMY, where they modulate dopaminergic transmission. The NAc, through its core and shell subdivisions, is a pivotal brain area associated with reward and pleasure processing, which has been shown to be regulated by serotonin through 5-HT1B and 5-HT2C receptors (81). Similarly, disruptions in serotonin levels within the AMY have been linked to decision-making, impulse control, and emotions such as anxiety and fear in the context of BADs (82, 83). Furthermore, serotonin produced by serotonergic neurons in the DRN/MRN projects to various brain regions, including the PFC (84, 85). In the PFC, 5-HT primarily exerts inhibitory effects on pyramidal neurons, thereby contributing to the prefrontal inhibition of potentially harmful behaviors (86, 87). Thus, it is evident that serotonin release in regions such as the NAc, AMY, and PFC influences decision-making, behavioral control, and emotional changes in BADs by modulating the DA system.

While serotonin is not a part of the subcortical dopaminergic regulatory system, it can exert influence over the DA system through direct or indirect modulation. Specifically, dysregulation of the serotonergic system may contribute to clinical manifestations such as behavioral inhibition and impulsivity in GD. One study found (88) a positive correlation between the severity of gambling problems in 10 males with GD and levels of 5-HT1B receptors in the VS and ACC. Furthermore, factors like increased anxiety and depression in individuals with GD suggest that 5-HT release in the serotonergic system may play a crucial role in alleviating negative emotions in patients (89). Previous research (90) investigated the availability of serotonin transporters (SERT) in individuals with GD compared to healthy controls and found no intergroup differences. However, a recent study (43) discovered a positive correlation between increased SERT activity in the PFC and impulsivity in elderly individuals with GD. Another study (91) further demonstrated that higher SERT binding potential in the prefrontal region was associated with imbalanced decision-making (leaning more towards habit-based control) in individuals with GD. These findings suggest that abnormal increases in SERT may also be one of the pathogenic mechanisms of BADs. Additionally, the levels of the serotonin metabolite 5-hydroxyindoleacetic acid (5-HIAA) in cerebrospinal fluid and the decreased activity of platelet monoamine oxidase (MAO) in blood (considered as a peripheral marker of 5-HT activity) (92–94) provide additional support for serotonin dysfunction in male gambling. In addition to findings related to gambling addiction, scholars have also discussed the significance of the serotonin system in IGD. It has been reported that prolonged exposure to electronic devices can lead to insufficient levels of vitamin D3 and melatonin in the body, resulting in gradual dysregulation of DA and serotonin neurotransmitter pathways in the brain, leading to addictive behaviors (95–97). Decreased levels of 5-HT have been found to be associated with the severity of internet gaming addiction and depressive mood, while regular exercise can increase 5-HT levels in the blood, alleviating negative emotions in individuals with IGD. Furthermore, a study (47) reported a correlation between decreased availability of 5-HT2A receptors in the temporal cortex and decreased availability of dopamine D2 receptors in the striatum in individuals with IGD. In addition to directly regulating DA release, evidence suggests that serotonin may modulate endorphins release in the HYP, potentially affecting GABA inhibition in the SNc and VTA, which could influence DA release in the NAc (80). This pathway may represent one of several mechanisms contributing to BADs. However, caution should be exercised in interpreting the stability and generalizability of the above research findings.

Collectively, serotonin dysregulation is another pathogenic mechanism involved in BADs, as it influences key neural circuits related to reward processing, decision-making, and impulse control, primarily through its interactions with dopaminergic, glutamatergic, and GABAergic systems. Previous pharmacological studies offer a theoretical foundation for investigating the neurobiological aspects of BADs and identifying key therapeutic targets. One such target is the SERT, which plays a pivotal role in regulating 5-HT reuptake. SERT’s function is to reabsorb 5-HT from the synaptic cleft back into presynaptic neurons, thereby maintaining a stable 5-HT level and regulating serotonergic signaling strength (98). Dysregulation of SERT activity can lead to either excessive or insufficient serotonergic transmission, contributing to behavioral and emotional dysregulation in BADs. Previous studies have found that selective serotonin reuptake inhibitors (SSRIs) such as fluoxetine, paroxetine, and citalopram are effective in reducing symptom severity, craving, and maladaptive decision-making in GD (99–101), IGD (102–105), CSBD (106–108), and compulsive shopping disorder (109). The 5-HT1A receptors (110, 111) are inhibitory receptors that, when activated on glutamatergic pyramidal cells and/or GABAergic interneurons in the PFC, stimulate DA release in the frontal cortex. Research has shown (112–114) that 5-HT1A receptor agonists, such as buspirone, can ameliorate anxiety and depressive symptoms during withdrawal from substance addiction (e.g., cocaine, alcohol, nicotine). A study in rodents (73) has also demonstrated that buspirone can improve decision-making in the rGT. The 5-HT1B receptors are other inhibitory receptors primarily located in the SNc, where their activation inhibits the release of neurotransmitters such as GABA, acetylcholine (ACh), and Glu, thereby modulating neural excitability and reward processing (115–118). Preclinical and clinical studies (119–121) have highlighted the role of the 5-HT1B receptors in managing depression and anxiety and their association with aggression and impulse control. Meta-chlorophenyl piperazine (mCPP) (122, 123), a metabolite of trazodone, is a mixed agonist for 5-HT1 and 5-HT2 receptors, particularly 5-HT1B receptors. In individuals with GD, mCPP administration has been reported to elicit subjective feelings of ‘excitement’ or arousal, whereas control subjects typically report aversive reactions. The 5-HT1D receptors (124, 125) primarily distribute across regions, including the caudate putamen, NAc, olfactory cortex, DRN, and locus coeruleus (LC) in the brain of rats. A study (126) reported that individuals with GD exhibited a reduced response to the selective 5-HT1D receptor agonist sumatriptan in terms of growth hormone release, whereas in the control group, growth hormone release increased. Furthermore, the 5-HT2A receptors (127, 128) are excitatory receptors, with a significant concentration of binding sites in prefrontal brain areas, including the cortical and hippocampal regions, basal ganglia, and olfactory tubercle. These receptors are involved in modulating glutamate release, synaptic plasticity, and emotional regulation. Present research (129–132) showed that 5-HT acting on the 5-HT2A receptors can enhance Glu release in cortical pyramidal cells and GABAergic inhibition in the AMY. A study (133) on the treatment of GD with a 5-HT2A receptor antagonist (nefazodone) revealed that within eight weeks after treatment, the scores for Yale Brown Obsessive Compulsive Scale adapted for Pathological Gambling (PG-YBOCS) and the Pathological Gambling Clinical Global Impression (PG-GCI) decreased by 37% compared to baseline levels. In a recent case report (134), researchers used a combination of fluoxetine and risperidone to treat GD patients and noted that three patients did not experience gambling thoughts and behaviors for a year and a half following treatment, suggesting that fluoxetine, as an SSRI medication, primarily acts on the SERT, while risperidone is an atypical antipsychotic medication that blocks the 5-HT2A receptors with high affinity and has limited selectivity for the 5-HT2C and DA receptors. Therefore, combining these two medications may be more effective in controlling addiction than using them individually, expanding the potential utility of 5-HT2A antagonists in managing addiction. The 5-HT2C receptors are primarily situated on postsynaptic serotonergic neurons that interact with GABAergic, glutamatergic, dopaminergic, and cholinergic neurons. For instance, stimulation of 5-HT2C receptors (135, 136) in the VTA increases the firing rate of GABAergic interneurons, leading to a decrease in the firing rate of dopaminergic neurons. Conversely, it has been reported (137) that 5-HT2C receptor antagonists can increase dopaminergic neurotransmission and DA levels in the NAc and PFC. Furthermore, studies (138–140) have found that when 5-HT from the MRN acts on the 5-HT2C receptors of GABAergic interneurons in the PFC, it enhances the inhibitory effects of these interneurons, thereby counteracting the inhibitory effects of GABA on pyramidal output neurons. This relief of inhibition leads to increased activity of pyramidal output neurons and increased excitability of glutamatergic neurons. An animal experiment (141) demonstrated that 5-HT2C receptor antagonists can disrupt maladaptive decision-making patterns in rats in the rGT, suggesting their potential role in modulating maladaptive behaviors, though the translational relevance to humans remains to be established. Similarly, a study (142) exploring the use of the antidepressant agomelatine (an M1/M2 agonist and 5-HT2C antagonist) in pathological gambling patients also indicated that the medication not only ameliorates anxiety and depressive symptoms in GD but also diminishes gambling thoughts and behaviors. Additionally, while the mechanisms of 5-HT2B receptors (143–146), 5-HT5A receptors (147) and 5-HT7 receptors (148, 149) in BADs remain unclear, prior research has demonstrated their involvement in substance addictions. For example, 5-HT2B receptors are implicated in regulating impulsivity and aggression, 5-HT5A receptors in circadian rhythm and mood regulation, and 5-HT7 receptors in cognitive flexibility and emotional processing, suggesting their potential as future pharmacological targets for BADs.

### 2.3 Endorphins

Endorphins play a pivotal role in the brain’s reward system, similar to the roles of DA and serotonin. In individuals with BADs, DA is believed to be associated (19, 22) with reward anticipation and prediction error signals, while serotonin (12, 150) is involved in impulse inhibition and behavioral control, particularly through its effects on the PFC and its modulation of subcortical structures such as the AMY and NAc. The endogenous opioid system (151–155) influences DA neurotransmission in the mesolimbic pathway extending from the VTA to the VS and is also involved in assigning hedonic value to rewards and integrating reward-related information to guide goal-directed decision-making and execution. Additionally, it contributes to the perception of pleasure and the experience of cravings while modulating responses to rewards and losses. The endogenous opioid peptide system (156, 157) consists of β-endorphins (highest content in the VTA, NAc and HYP) (158–160), enkephalins (highest content in the VTA, NAc, SNc, HYP, striatum and hippocampus) (161–163), dynorphins (highest content in the PFC, SNc, striatum and central AMY) (161, 164–166), and nociceptins (highest content in the PFC and VTA) (167, 168). These peptides exert their effects through μ-opioid receptors (MOR), δ-opioid receptors (DOR), κ-opioid receptors (KOR) and nociceptin opioid peptide receptors (NOPR). These receptor systems are typically expressed at elevated levels in brain regions responsible for processing emotions, rewards, and aversions, such as the VTA, NAc, ACC, HYP, AMY and insula. β-endorphins (169) can bind to MOR and DOR, with a higher affinity for MOR, while enkephalins (170) have a stronger affinity for DOR than MOR and KOR. Dynorphins (157, 171, 172) have the highest affinity for KOR but can also bind to MOR and DOR within physiological ranges. Nociceptins (173, 174) specifically bind to NOPR and have very low affinity for other opioid receptors.

The role of endorphins in addiction appears to vary depending on their binding to different receptors (175–177). Ligands targeting MOR and DOR receptors may be associated with rewarding effects and emotional regulation, while ligands for KOR receptors may be linked to aversive effects. The function of NOPR receptor ligands in BADs remains uncertain at present. However, preclinical studies suggest that (178) NOPR activation may play a role in stress modulation and the attenuation of reward-seeking behaviors, indicating potential therapeutic implications for BADs. Previous studies (80, 179–181) have found that gambling or gambling-like activities (i.e., horse racing, slot machines, etc.) can trigger the release of endorphins, particularly in the NAc and VTA, which are key regions in the brain’s reward circuitry. Simultaneously, the increased availability of MOR in the shell, caudate nucleus, and globus pallidus is associated with presynaptic DA synthesis capacity, implying that the release of endorphins not only directly augments DA release but also, through MOR activation, inhibits the inhibitory neurotransmitter GABA in the VTA, thereby facilitating DA release in the NAc (182). Furthermore, it has been observed (183) that endorphins in the NAc increase in response to DA activation, suggesting a potential feedback loop between the dopaminergic and opioid systems. Conversely, excessive activity of the endorphin system can modulate dopaminergic function (184).

Up till now, researchers have investigated the effects of specific opioid receptor agonists and antagonists in the addiction process (185). On one hand, opioid agonists enhance DA release in the NAc, significantly increasing pleasure and leading to orgasmic-like cravings. On the other hand, opioid receptor antagonists inhibit DA release in the NAc and the ventral pallidum by disinhibiting GABAergic inputs to dopaminergic neurons in the VTA, suppressing the excitatory and craving-related effects associated with BADs. These findings may be important considerations for the future development of treatments for BADs, particularly in targeting the opioid-dopaminergic interactions that drive craving and reinforcement mechanisms. Opioid antagonists, such as naltrexone and nalmefene, primarily target MOR, DOR, and KOR (186), albeit with a lower affinity for DOR and KOR. By blocking MOR, these antagonists reduce the rewarding effects of addictive behaviors and substances, while their effects on DOR and KOR may contribute to mood stabilization and stress regulation. A recent meta-analysis of drug treatments for GD investigating the findings from four randomized controlled studies on naltrexone and nalmefene (187–190) found that opioid antagonists can improve the severity of GD in the short term. However, there is currently insufficient evidence to determine their efficacy in addressing the psychological symptoms of gambling, such as impulsivity and craving, or their long-term effectiveness in preventing relapse. In this regard, a study by Kim et al. (187) found that naltrexone was more effective in treating individuals reporting severe impulsive behaviors compared to those with low impulsive behaviors. Furthermore, concerning the dosage aspect of pharmacological treatment, Grant et al.’s research indicated that high doses of nalmefene (40mg/day) were significantly more effective than placebo in treating GD symptoms (188, 190). However, naltrexone (189) was found to be effective at lower doses (50mg/day) with fewer adverse effects. Additionally, despite the absence of large-scale randomized controlled trials, naltrexone (191, 192) has demonstrated preliminary efficacy in reducing CSBD symptoms and impulsive behaviors, while nalmefene (193, 194) has demonstrated positive effects on addiction symptoms and behaviors related to internet pornography addiction. Presently, the mechanism of action of NOPR in BADs is still unclear. Nevertheless, a human study (178) employing PET imaging to assess changes in NOPR binding using radiolabeled nociceptin identified increased NOPR levels in participants with cocaine use disorder, particularly in the midbrain, VS, and cerebellum, providing insights for further exploration of the role of NOPR in BADs.

### 2.4 Gamma-aminobutyric acid

GABA, a pivotal inhibitory neurotransmitter in the central nervous system of mammals (195, 196), reduces neuronal excitability. It is primarily synthesized from Glu via the enzyme glutamic acid decarboxylase (GAD), with pyridoxal phosphate (the active form of vitamin B6) as a cofactor. Like endorphins, serotoninergic and glutamatergic neurons in the DRN can modulate GABA input to the SNc, consequently impacting DA release (197). Additionally, GABA (198–201) can also exert its effects by projecting from the ventral pallidum, VTA GABAergic interneurons and the medial spiny GABAergic neurons of the NAc, inhibiting DA release from the mesolimbic system through GABAA and GABAB receptors. It is important to recognize that although both Glu and GABA can influence DA release, their effects on DA are inversely related (202). Therefore, GABA plays a pivotal role in the reward process associated with BADs, acting as a critical intervention point and can directly impact DA release and indirectly modulate DA release by influencing Glu projections within the NAc.

In the past, research on GABA has primarily explored substance addiction, particularly its role in alcohol addiction (203, 204). Similar to substance addiction, there are some similarities in the neurobiology of BADs. With the advancement of experimental techniques, scholars have gradually started investigating the specific manifestations of GABA in BADs. For instance, a study (205) utilizing magnetic resonance spectroscopy (MRS) technique found that in male individuals with GD, the discounting of small immediate rewards in the dorsal anterior cingulate cortex was negatively correlated with GABA, while the discounting of larger delayed rewards was negatively correlated with the ratio of GABA/glutamate-glutamine (Glx) in the dorsolateral PFC. Mick et al. (206) used [11C]Ro15-4513 as a radioligand for GABAA receptors and detected increased GABA binding in the right hippocampus of individuals with GD. They also found a direct correlation between increased GABA binding to GABAA in the AMY and impulsivity (negative urgency) related to emotional factors in the GD group. Furthermore, Chowdhury et al. (207) found weaker GABAA receptor activity but higher Glu receptor activity in the primary motor cortex (M1) of problem gamblers compared to non-gamblers and high-risk gamblers, suggesting an imbalance in excitatory and inhibitory neurotransmission in this region. Additionally, the compromised response inhibition ability of individuals with gambling addiction correlated with reduced GABAA receptor activity in M1, suggesting that decision-making and impulsive behaviors in those with gambling addiction are influenced not only by GABAA receptor activity within the reward circuitry but also by alterations in glutamate-mediated neurotransmission. In a study (208) investigating internet and smartphone addiction, it was observed that addiction severity, as well as symptoms of depression and anxiety, correlated with elevated GABA levels in the ACC. After nine weeks of cognitive-behavioral therapy, GABA levels tended to normalize. These findings contrast somewhat with research (209) on substance addiction, which may be related to elevated GABA levels leading to reduced ACC function (210). However, it is essential to note that the sample size of this study was small, and further validation of these findings is needed.

In summary, the dysregulation of the GABA system emerges as a potential target for pharmacological intervention in BADs, with a primary focus on GABAA and GABAB receptors. Previous animal studies (74, 211) showed that the intracerebral injection of a combination of GABAA receptor agonist (muscimol) and GABAB receptor agonist (baclofen hydrochloride) results in receptor inactivation in the PFC, ACC, and HYP regions. This inactivation weakens rodents’ ability to differentiate between winning and losing outcomes in a rGT, leading to a preference for disadvantageous options and reduced selection of optimal choices. Moreover, this combination’s effects have been observed to induce insular cortex inactivation in rats during a radial arm maze test, prompting risky decision-making behaviors (212). In clinical investigations, substance addictions such as alcohol addiction (213), nicotine addiction (214) and heroin addiction (215) have been found to be closely related to GABAA and GABAB receptors. In BADs, only one experimental study (62) indicated that compared to healthy volunteers, levodopa reduces the availability of GABAA receptors in the PFC and insular regions of problem gamblers seeking treatment, leading to decreased GABA release and a loss of inhibitory control, suggesting that dysfunctional DA regulation of GABA release may contribute to GD. While these findings are promising, caution is necessary due to limitations such as small sample sizes and insufficient control of confounding factors. Further rigorous research is required to confirm these results and establish their clinical relevance. Nonetheless, these findings offer new strategies for the treatment of BADs in the future.

### 2.5 Glutamate

In the reward circuitry of BADs, not only are DA projections from the VTA to the PFC (18, 216), serotonin projections from the MRN to the NAc (81), and GABA projections from the NAc to the ventral pallidum involved (198, 217), but Glu projections from the PFC to the NAc also play an important role (218, 219). Glu projections contribute to changes in cognitive functioning, especially cognitive flexibility, which is essential for adapting to new situations and modifying behavior. These projections enable individuals to consciously resist impulses and form new associations between stimuli (e.g., gaming) and behavioral responses, linking them to unconditioned responses such as reward or punishment. Glu is a naturally occurring amino acid and a fundamental component of proteins. It is the most widely distributed excitatory neurotransmitter in the brain (220). Glu and glutamine (Gln) can be interconverted through the action of glutamine synthetase, establishing a “Glu-Gln cycle” between glial cells and neurons. This cycle allows for the continuous recycling and regeneration of Glu (221). Maintaining the balance of Glu between synapses and glial cells is crucial for the PFC to effectively regulate the reward-sensitive NAc. This balance ensures proper excitatory signaling and prevents excessive Glu activity, which could dysregulate reward processing and decision-making. Insufficient Glu levels in glial cells can lead to increased Glu release at synapses, significantly enhancing DA release in the NAc (221). When the Glu pathway is compromised, individuals may become more motivated by short-term rewards at the expense of long-term objectives. This imbalance in decision-making is associated with impaired cognitive control and heightened impulsivity, which are characteristic of BADs.

Current theories and empirical evidence suggest (219, 222, 223) that Glu from the PFC regulates DA levels through multiple pathways. While direct glutamatergic projections from the PFC to VTA are excitatory, the overall relationship between cortical Glu and DA transmission is complex and can involve inhibitory circuits. Glu from the PFC and/or AMY can modulate reward-driven behavior by affecting the responsiveness of DA cells in the VTA-NAc pathway, thus influencing DA’s reward-focused effects on decision-making. It has been reported (218) that compared to the ADHD group, the ADHD+IGD group showed decreased levels of Gln in the right PFC, which may be associated with the increased DA levels caused by excessive online gaming. Additionally, prolonged and excessive exposure to electronic games and other digital entertainment that provide immediate rewards can downregulate DA and Glu receptors in the NAc, resulting in symptoms such as tolerance, withdrawal and compulsive seeking of stimulation (48, 224). Furthermore, in a study of male samples with GD (205), the researchers reported a negative correlation between baseline Glx levels in the dorsal ACC and the severity of gambling. In another study (225), compared to 10 healthy males, 10 male pathological gamblers showed an increasing trend in Glu and aspartate levels in cerebrospinal fluid.

To summarize the contents described in this section, Glu can be considered as one of the essential elements for understanding the mechanisms underlying BAD’s formation from a psychopharmacological perspective. A number of pharmacologic studies (226) also provide evidence for abnormal Glu function in individuals with BADs, revealing that targeted interventions on Glu receptors can positively alleviate BAD’s symptoms. Glu exerts its effects through two different types of receptors (220), namely, metabotropic glutamate receptors (mGluRs) and ionotropic glutamate receptors (AMPA, NMDA and Kainate). Specifically, N-acetylcysteine, which acts on inhibitory mGluR2/3 receptors, first increases extracellular Glu levels through the cystine-glutamate exchanger, which then stimulates mGluR2/3 receptors, thereby effectively reducing Glu synaptic release (227–229). When extracellular Glu levels are restored in the NAc, there is a certain inhibitory effect on cravings and impulsive behavior associated with addiction. Research (230) in rats has shown that N-acetylcysteine effectively reduces reward-seeking behavior. Clinical studies (231, 232) on GD have shown a significant reduction in gambling severity with active N-acetylcysteine treatment, a change that largely persists during the double-blind withdrawal phase. A recent case study involving a 19-year-old male with IGD (233) reported that after one month of N-acetylcysteine treatment at a dosage of 600mg twice daily, the patient experienced a significant decrease in cravings for gaming, consistent with findings from studies on GD and substance use disorders. Although all of the aforementioned clinical studies are preliminary and involved relatively small sample sizes, the consistent anti-addiction properties of N-acetylcysteine provide compelling evidence that this medication may be an effective adjunct in treating BADs. NMDA receptors have also been found to play a role in animal experiments (234). Blocking NMDA receptors (but not AMPA receptors) with the antagonist MK-801 hydrochloride reduces sensitivity to delayed reinforcement, uncertain reinforcement, and the amount of reinforcement in rats in operant conditioning chambers. In clinical trials, memantine, a non-competitive NMDA receptor antagonist, has shown promise in reducing impulsive behaviors and improving cognitive flexibility in individuals with GD (235). Researchers have reported an average reduction of 35.1% in PG-YBOCS scores compared to baseline and significant improvement in cognitive flexibility during the intra-dimensional/extra-dimensional (ID/ED) set shift task. These findings may be attributed to memantine’s modulation of glutamatergic neurotransmission in the PFC, reducing impulsive behaviors. Additionally, bupropion, which also acts as a non-competitive NMDA receptor antagonist, has been effective in reducing the severity of GD in patients and diminishing cravings for video games in individuals with IGD (103, 236). In addition to binding to NMDA receptors, bupropion can also interact with AMPA receptors, Kainate receptors, 5-HT3 receptors, and MOR, inhibiting DA and NE reuptake, thereby exhibiting a relatively complex mechanism of action in addiction. Case reports have shown that other NMDA receptor antagonists, such as acamprosate (237) and amantadine (238), may also have therapeutic effects on BADs. The AMPA receptor antagonist carbamazepine, as an antiepileptic drug, has been shown to effectively treat patients with GD by binding to AMPA receptors. By binding to AMPA receptors, carbamazepine reduces excitatory glutamatergic signaling, which may help regulate impulsive and compulsive behaviors associated with GD. In an open-label study by Black et al. (239), five individuals with GD received extended-release carbamazepine treatment for eight weeks, and the findings showed significant reductions in the participants’ PG-YBOCS scores and Gambling Severity Assessment Scale (GSAS) scores, consistent with previous case reports on the use of carbamazepine for GD (240). Topiramate, another antiepileptic drug, has multiple mechanisms of action (241), including the inhibition of AMPA receptors, mGluR5 receptors, and activation of GABAA receptors. In a case study from Brazil (242), a 57-year-old elderly woman with bipolar disorder and GD showed significant improvement in her gambling behavior when topiramate was added to her lithium carbonate treatment at a dose of 200mg/day and reported no cravings for gambling after two months of combination therapy. Moreover, topiramate has demonstrated positive effects in the treatment of CSBD (243) and compulsive buying disorder (244). Currently, studies have identified several glutamate transporters as key regulators of glutamatergic signaling in substance addictions. These include glutamate transporter-1 (GLT-1)) (245), which facilitates Glu reuptake into glial cells; excitatory amino acid transporters 1 and 3 (EAAT-1/3) (246, 247), which regulate synaptic Glu clearance; and vesicular glutamate transporters 1 and 2 (VGLUT-1/2), which mediate Glu storage and release from presynaptic neurons (248, 249).

### 2.6 Norepinephrine

NE, a catecholamine synthesized from DA by dopamine β-hydroxylase (DBH), is primarily released under stress to enhance individual excitability (186, 250). Due to its comprehensive role in arousal and attention regulation, NE from the LC is increasingly associated with addiction through its projections to the nucleus tractus solitarius (NTS)-NAc area (251). In individuals with GD, particularly male patients, excitement is often identified as a significant factor contributing to gambling. Previous studies (252, 253) have found that problem gamblers exhibit significantly higher levels of NE in their blood, urine, and cerebrospinal fluid during gambling compared to control groups. Additionally, a study investigating (179) psychological changes in players during a pachinko game revealed that NE, β-endorphin and DA levels were elevated during the initiation and winning phases compared to baseline levels and that gambling behavior was associated with increased heart rate and respiratory rate (254). These findings suggest a connection between gambling behavior and the autonomic arousal system targeted by NE. Recent research based on electroencephalography (EEG) has shown (255) that the enhancement of the P300 component during a two-choice gambling task called a two-armed bandit was dependent solely on the exploration phase, indicating that NE, rather than DA, plays a crucial role in triggering exploratory decisions. Interestingly, studies on IGD (256, 257) have found that compared to control groups, individuals with IGD had lower levels of NE during resting states. Furthermore, excessive playing of online games leads to decreased peripheral adrenaline and NE levels over time, altering autonomic regulation and increasing anxiety levels in adolescents. These findings contrast with the elevated levels of NE associated with GD (254), potentially due to the chronic stress stimulation caused by prolonged internet gaming (21, 258), which leads to adaptive responses such as receptor downregulation in the central nervous system. Chronic stress and prolonged gaming lead to a shift in behavioral control from goal-directed behavior, mediated by the PFC, to habitual control, which involves the dorsal striatum. This transition is driven by repetitive actions and changes in neurotransmitter regulation.

Interests in the role of NE in BADs are resurging. Early clinical and preclinical studies provide valuable insights for potential BAD treatments. DBH can convert DA to NE, and in a rodent experiment, it was found (259) that disulfiram, a DBH inhibitor, improved the performance of rats with disadvantageous strategies in the rGT, with a decrease in NE and an increase in DA observed in the striatum. Case reports have also suggested (260, 261) that disulfiram may reduce gambling cravings in individuals with GD. However, larger clinical trials are needed to confirm its efficacy and tolerability. Disulfiram’s effects may be mediated by its inhibition of DBH, which shifts the NE-DA balance in favor of DA. This mechanism could reduce stress-induced arousal and impulsivity, which are key drivers of gambling behavior. Further, the involvement of norepinephrine transporter (NET) in BADs has also been reported. Atomoxetine, as a NET blocker, has been shown to improve decision-making in male and female rats in the rGT by increasing synaptic NE levels (262). NE interacts with other neurotransmitters, such as DA and Glu, to regulate reward sensitivity and cognitive control. NE modulates DA release in the NAc, influencing the reward system’s sensitivity to stimuli. Additionally, in adolescents with comorbid ADHD and IGD, treatment with atomoxetine for 12 weeks resulted in a significant reduction in impulsivity and severity of internet gaming addiction (77). Other studies have revealed the important roles of α-2 adrenergic receptors in preclinical studies (262). Guanfacine, an α-2 adrenergic receptor agonist, which diminishes NE neuron firing by acting on autoreceptors, selectively enhanced decision-making abilities in risk-prone male rats and optimally performing female rats. In clinical research (263), the atypical stimulant modafinil was found to potentially reduce gambling cravings, impulsive behavior and risky decision-making in individuals with GD, which can be mediated through the stimulation of α-2 adrenergic receptors, inhibition of GABA release, elevation of extracellular Glu levels, weak inhibition of DAT or stimulation of HYP orexin neurons. Additionally, compared to healthy controls, male pathological gamblers showed an increased growth hormone response to the α-adrenergic receptor agonist clonidine (264). Furthermore, a study using functional magnetic resonance imaging (fMRI) (265) demonstrated differential activation in the AMY between individuals with GD and those without GD in response to yohimbine, an α-2 adrenergic antagonist. The β-adrenergic antagonist propranolol (266) has been shown to reduce compulsive gambling behavior in rodents in a slot machine task. Similarly, in a human gambling task (267), propranolol treatment did not result in significant changes in subjective state or mood compared to placebo. However, it did selectively alter decision-making in volunteers, specifically attenuating the processing of potential losses, indicating a reduced sensitivity to punishment cues.

### 2.7 Other neurotransmitters and neurotrophic factors

The neurobiological mechanisms underlying BADs involve the participation of multiple neurotransmitters. In addition to the neurotransmitters discussed above, such as DA, 5-HT and Glu, there are several other promising neurotransmitters and neurotrophic factors that may play a role in the pathogenesis of BADs, although current research in this area is still limited.

#### 2.7.1 Corticotropin-releasing factor

It has been implicated in the research on rGT in rodents. The overexpression of CRF receptor 1 in the AMY of female rats has been found to be associated with increased risk-taking behavior (268). Additionally, compared to male rats, CRF receptor 1 antagonist (antalarmin) may be more effective in improving decision-making in female rats. A study involving Korean adolescent boys (269) also confirmed the association between polymorphisms in the CRF receptor gene and IGD, and it was found that individuals carrying the AA genotype and the A allele of rs28364027 (CRF1 gene) were more prone to IGD. Furthermore, several studies examining cortisol levels in BADs (270–275) indirectly suggest that abnormalities in the CRF system may underlie changes in decision-making patterns, cravings and stress responses associated with anxiety and emotional states.

#### 2.7.2 ACh

Previous animal studies have reported (276) that muscarinic receptor antagonists (i.e., scopolamine) can improve decision-making patterns in the rGT task, increasing rats’ preference for advantageous options while reducing their selection of risky options. However, contradictory results have also been reported (277). Nicotinic receptor antagonists (such as mecamylamine) (276), while not affecting decision-making, were shown to be associated with reduced impulsive behavior in rats. On the other hand, another relevant study in mice (278) suggests that neuronal nicotinic acetylcholine receptors may play a crucial role in the decision-making process. Studies by Montag et al. (279) and Jeong et al. (280) have both found an association between rs1044396 (CHRNA4 gene, encoding the α-4 subunit of the nicotinic ACh receptor) and IGD. ACh receptors also play a role in BADs by modulating the DA pathway, and increased cholinergic tone may be one of the factors affecting decision-making and impulse control in BADs.

#### 2.7.3 Oxytocin

OXT has been found to primarily exert its effects through the oxytocin receptor (OXTR) in research related to addiction. Studies (281, 282) have indicated that rs2254295 and rs2268498 can modulate the function or expression of the OXTR gene, and individuals with the TT genotype have a lower risk propensity compared to participants with the CT and CC genotypes. Young male participants who inhaled intranasal OXT during the IGT exhibited a significant reduction in risk-taking behavior during the decision-making process under uncertainty. In regards to IGD, it has also been found (283) that male carriers of the TT genotype have lower levels of internet addiction. Furthermore, the level of plasma OXT was found to increase when individuals with CSBD, particularly those with problematic pornography use, were exposed to positive social stimuli (284). Therefore, the hypothesis that OXT may be a potential protective factor in BADs appears compelling.

#### 2.7.4 Orexin

The role of the OX system (285) in regulating motivation and reward-seeking behavior in substance addiction has been well-established. Animal studies on BADs (286, 287) revealed that rats with a preference for high-reward outcomes exhibited increased orexin receptor 1 (OX1R) expression in the HYP and hippocampus. OX1R may be involved in impulsive behavior mediated by the HYP and hippocampus and in the selection of positively reinforced choices based on varying intensity and probability in the rGT task. Choi et al. confirmed (271) an increase in plasma OX levels in adolescents with IGD, indicating its potential involvement in IGD. OX may participate in the formation of BADs through its interaction with GABA and DA.

#### 2.7.5 Leptin

Leptin has been suggested as a potential modulator of reward-related behaviors by regulating satiety and possibly influencing addictive behaviors through the mesolimbic reward pathway (288). Previous studies on patients with substance use disorders (alcohol, cocaine) (289, 290) have reported a positive correlation between leptin levels and craving. A recent study on healthy participants performing the IGT (291) demonstrated that individuals with higher leptin levels had worse performance on the IGT, while another study (292) revealed no relationship between leptin and craving in male IGD and GD. Overall, leptin is believed to be involved in the mechanisms underlying the formation of BADs through its interaction with the hypothalamic-pituitary-adrenal (HPA) axis.

#### 2.7.6 Melatonin

It is a well-known regulator of various signaling pathways and biological rhythms (293). It has been reported that modulating melatonin can alter the behavior and physiological functions of individuals with substance addiction (294, 295). However, there is limited research on melatonin in regards to BADs, with only one study (142) indicating that individuals with GD showed improvements in addiction severity, anxiety and depression after treatment with agomelatine (a melatonin M1/M2 receptor agonist).

#### 2.7.7 Brain-derived neurotrophic factor

BDNF is highly expressed in limbic structures and the cerebral cortex and plays a crucial role in learning, memory, and reward-related processes (296). Several studies (297–300) have reported that BDNF levels positively correlate with the severity of GD or IGD in individuals. The increase in BDNF in BADs may be associated with alterations in DA transmission in the VTA and NAc. However, some studies (301, 302) have found no correlation between BDNF and BADs, suggesting the need for further investigation in longitudinal experiments.

#### 2.7.8 Glial cell line-derived neurotrophic factor

GDNF is a neurotrophic factor involved in the development of dopaminergic neurons (303), and its role in reward mechanisms has been demonstrated in animal models of substance addiction (304). Current research (305) suggests that the GDNF gene variant rs2973033 is significantly associated with GD. Furthermore, it has been found (306) that plasma levels of GDNF in individuals with IGD are significantly lower compared to healthy controls and are negatively correlated with addiction severity.

Research on the mechanisms of neurotransmitters and neurotrophic factors in BADs remains limited, requiring validation through large-scale multicenter studies. However, there are promising indications that they may play a role in BADs by modulating DA, serotonin or GABA pathways in the reward circuitry. These mechanisms are integral to BADs and may present a new hope for the treatment of BADs for clinicians.

## 3 Emerging technologies: potential applications in BADs research

In recent years, emerging technologies have provided novel perspectives for research on BADs, particularly through the integration of CRISPR gene editing technology and neuroimaging, significantly advancing our understanding of neurotransmitter crosstalk mechanisms. CRISPR technology, through precise gene expression regulation, has revealed molecular mechanisms of dopaminergic neurons in the NAc (307). For instance, using CRISPRa and CRISPRi tools, researchers discovered that bidirectional regulation of Egr3 and Nab2 in D1-MSNs and D2-MSNs is crucial for reward processing and impulse control, while the innovatively developed light-sensitive Opto-CRISPR-KDM1a system not only achieved dynamic regulation of these genes but also revealed the key role of histone lysine demethylase in drug addiction (308). Through precise CRISPR-mediated regulation of COMT gene expression, researchers found that MB-COMT plays a crucial role in PFC dopamine metabolism, and this regulation directly influences cognitive control and reward processing through its balance with the GABAergic system (309). Neuroimaging studies further demonstrate significant dysfunction in emotion regulation networks among BADs patients, particularly abnormal activities in the PFC, striatum, and limbic system. Network-based fMRI analysis reveals that substance and BADs share functional alterations in prefrontal-striatal circuits, closely associated with dopaminergic system dysregulation (310). Recent studies have found that drug abuse “hijacks” the brain’s reward system, leading to enhanced dopaminergic neuronal ensemble activity in the NAc and disrupted responses to natural rewards (307). Complementary EEG studies have revealed characteristic changes in beta-band power and brain network connectivity in BADs patients, closely related to trait and behavioral impulsivity (311). Non-invasive neuromodulation techniques such as rTMS have shown promising results in treating GD, while the integration of neuroimaging markers enables more accurate prediction and monitoring of treatment responses (312). The integration of these emerging technologies has not only deepened our understanding of the neurobiological mechanisms of BADs but also established a solid scientific foundation for developing personalized treatment approaches and multi-target intervention strategies.

## 4 Conclusions and future perspectives

**Figure 1:**
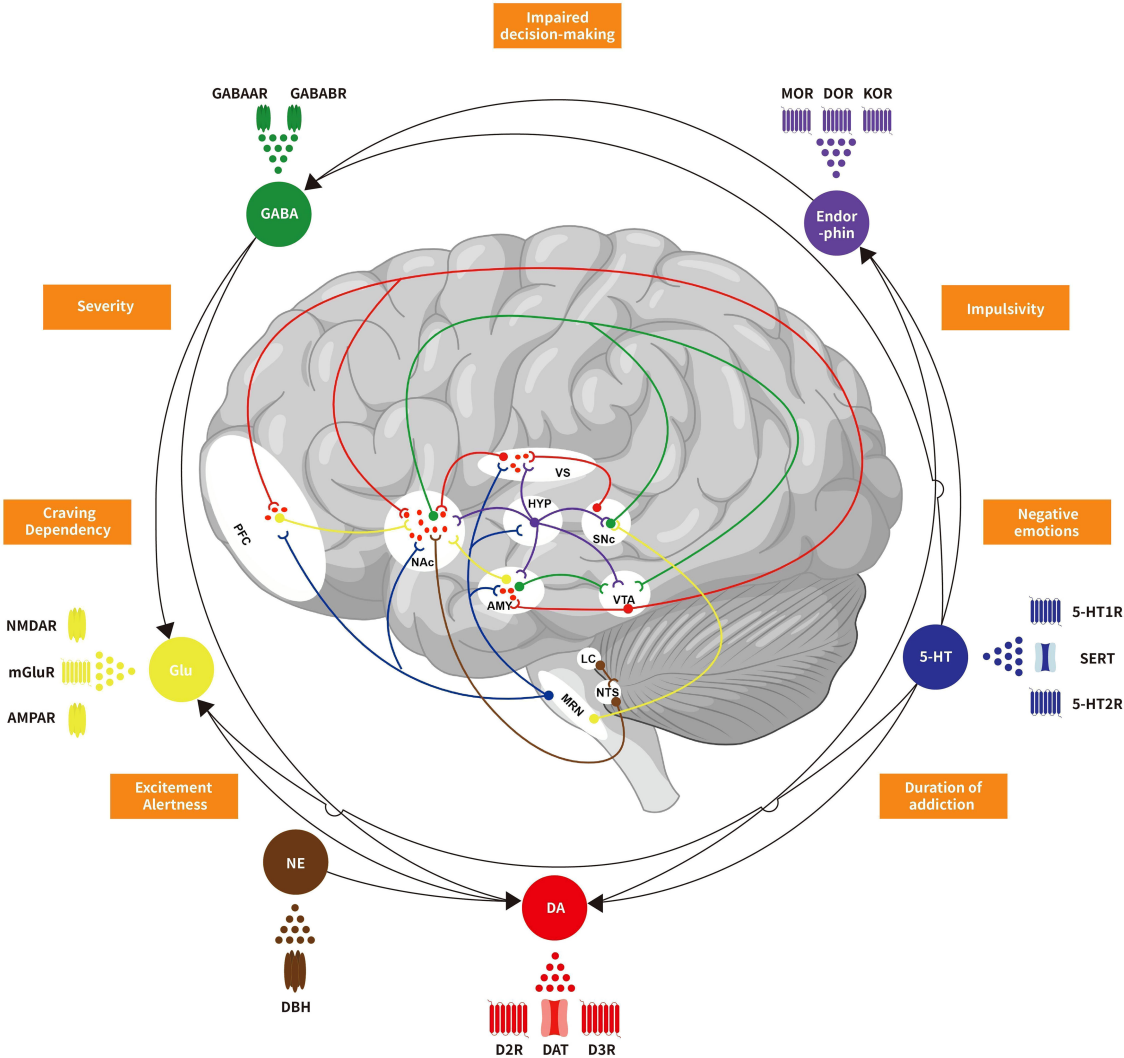
Schematic representation of neurotransmitters crosstalk and regulation in behavioral addiction reward circuit. Dopamine, serotonin, endorphins, GABA, glutamate, norepinephrine, and other neurotransmitters collectively form a complex regulatory network in the reward circuitry of behavioral addictive disorders. These neurotransmitters interact and interfere with each other, influencing not only biological behaviors but also closely associated with the occurrence and development of addiction. VTA, Ventral tegmental area; NAc, Nucleus accumbens; VS, Ventral striatum; PFC, Prefrontal cortex; AMY, Amygdala; SNc, Substantia nigra pars compacta; HYP, Hypothalamus; MRN, Median raphe nuclei; LC, Locus coeruleu; NTS, Nucleus tractus solitariu; DA, Dopamine; 5-HT, 5-hydroxytryptamine; GABA, Gamma-aminobutyric acid; Glu, Glutamate; NE, Norepinephrine; D2R, Dopamine D2 receptors; D3R, Dopamine D3 receptors; DAT, Dopamine transporters; 5-HT1R, 5-HT1 receptors; 5-HT2R, 5-HT2 receptors; SERT, serotonin transporters; MOR, μ-opioid receptors; DOR, δ-opioid receptors; KOR, κ-opioid receptors; GABAAR, GABAA receptors; GABABR, GABAB receptors; mGluR, Metabotropic glutamate receptors; NMDAR, N-methyl D-aspartic acid receptors; AMPAR, Alpha-amino-3-hydroxy-5-methyl-4-isoxazole propionic acid receptors; DBH, Dopamine-beta-hydroxylase.

**Figure 2:**
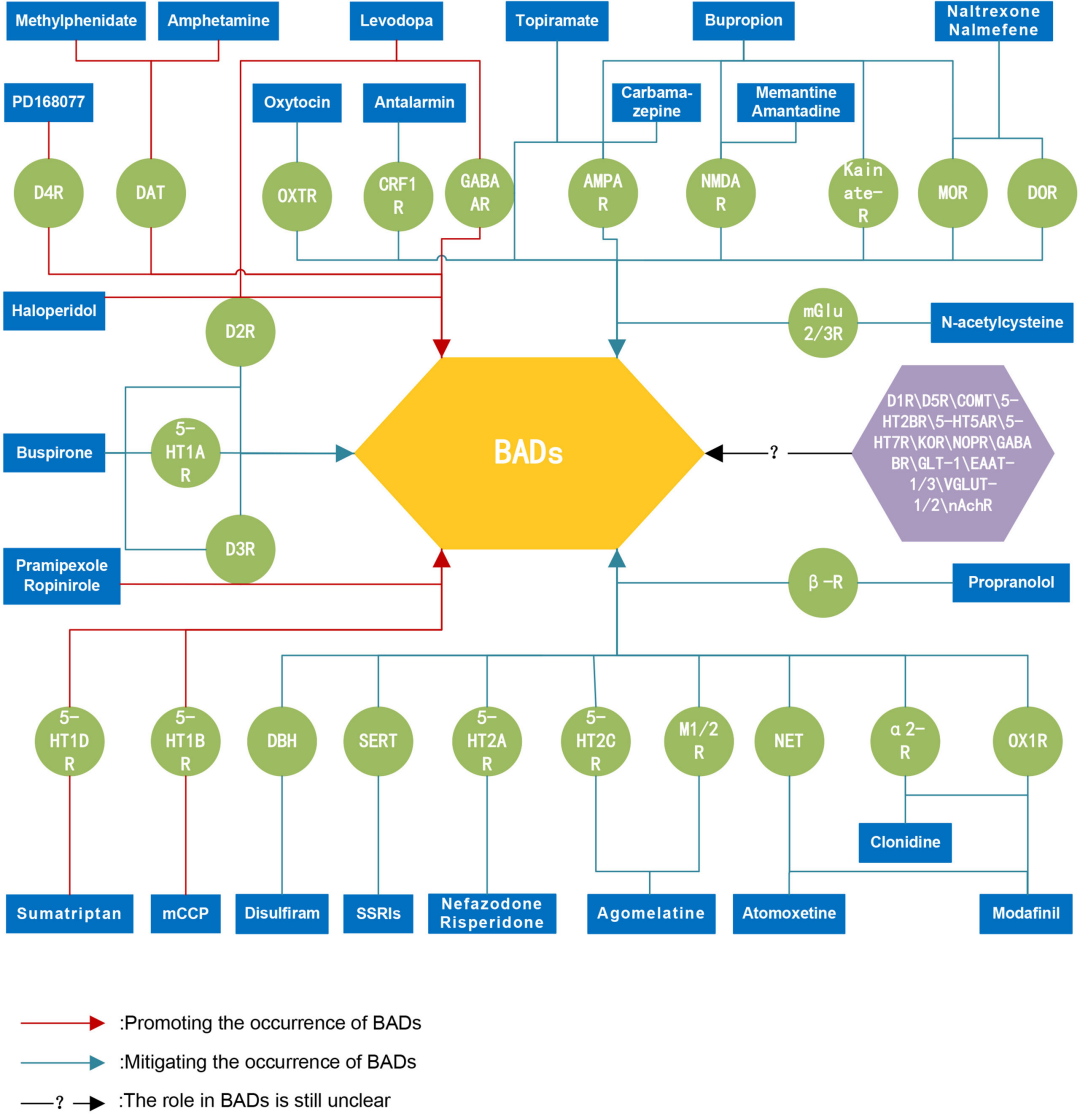
Schematic representation of potential pharmacological targets for the treatment of BADs. There are several important neurotransmitter targets involved in the occurrence and development of behavioral addictive disorders. These targets hold potential therapeutic prospects, and targeted "neurotransmitter therapies" may provide overall treatment benefits for individuals with behavioral addiction disorders without causing apparent damage to the body's physiological systems. BADs, behavioral addictive disorders; D1R, Dopamine D1 receptors; D2R, Dopamine D2 receptors; D3R, Dopamine D3 receptors; D4R, Dopamine D4 receptors; D5R, Dopamine D5 receptors; DAT, Dopamine transporters; COMT, catechol-O-methyltransferase enzyme; 5-HT1AR, 5-HT1A receptors; 5-HT1BR, 5-HT1B receptors; 5-HT1DR, 5-HT1D receptors; 5-HT2AR, 5-HT2A receptors; 5-HT2BR, 5-HT2B receptors; 5-HT2CR, 5-HT2C receptors; 5-HT5AR, 5-HT5A receptors; 5-HT7R, 5-HT7 receptors; SERT, serotonin transporters; MOR, μ-opioid receptors; DOR, δ-opioid receptors; KOR, κ-opioid receptors; NOPR, Nociceptin opioid peptide receptors; GABAAR, GABAA receptors; GABABR, GABAB receptors; mGlu2/3R, Metabotropic glutamate 2 and 3 receptors; NMDAR, N-methyl D-aspartic acid receptors; AMPAR, Alpha-amino-3-hydroxy-5-methyl-4-isoxazole propionic acid receptors; Kainate-R, Kainate receptors; GLT-1, Glutamate transporters-1; EAAT-1/3, Excitatory amino acid 1 and 3 transporters; VGLUT-1/2, Vesicular glutamate 1 and 2 transporters; DBH, Dopamine-beta-hydroxylase; NET, Norepinephrine transporters; α2-R, alpha-2 receptors; β-R, beta receptors; CRF1R, Corticotropin-releasing factor 1 receptors; M1/2R, Muscarinic acetylcholine 1 and 2 receptors; nAchR, Nicotinic acetylcholine receptors; OXTR, Oxytocin receptors; OX1R, Orexin 1 receptors; SSRIs, selective serotonin reuptake inhibitors; mCCP, Meta-chlorophenyl piperazine.

Advances in neuroscience have improved our understanding of how neurotransmitters contribute to the development of BADs. This work provides an overview of the neurobiology of BADs, focusing on the complex interplay of multiple neurotransmitters. The phenotypes of addiction arise from disruptions in neurotransmitter and neurotrophic factor expression and function. DA, a key neurotransmitter in the reward pathway, plays a central role in the mechanisms of BADs by modulating reward sensitivity, motivation, and reinforcement learning. DA interacts with other neurotransmitters, including serotonin, endorphins, GABA, Glu, NE, and neuropeptides such as CRF, ACh, OXT, and OX. Additionally, neurotrophic factors like BDNF and GDNF contribute to synaptic plasticity and neuroadaptations associated with BADs (see **Figure 1**). The interactions among these neurotransmitters influence biological behaviors such as reward processing, impulsiveness, and stress responses, which are closely linked to the onset and progression of BADs. Addiction is increasingly recognized as a chronic brain disorder, and evidence suggests it requires similar attention and treatment as other medical conditions. Neuroimaging and psychopharmacological investigations have identified anomalies in critical neurotransmitter targets among individuals with addiction. While these findings offer novel insights, further research is needed to validate their potential for treating BADs (see **Figure 2**). Despite substantial progress in the neurobiology of BADs and the preliminary efficacy and safety of pharmacological treatments for BADs, our understanding of the neurobiological mechanisms underlying their clinical features remains limited. To fully grasp the complexities of neurotransmitter actions in the brain, further research is needed to explore their interplay, regulation and how they ultimately drive behavior. Additionally, while there are some similarities in the neurobiology of different BADs, differences also exist. Therefore, future investigations should aim to delineate both the commonalities and unique neurobiological aspects inherent to various types of BADs, which could hold promise for identifying novel targets that could lead to more precise and personalized strategies for preventing and treating BADs. Finally, as neuroscientists and psychiatrists dedicated to addiction research, we advocate for the global scientific community to reconsider the prevailing dopamine-centric dedicated in BADs research. Leveraging advanced methodologies, such as genetic molecular localization, advanced imaging modalities, high-throughput single-cell analyses, and computational systems biology, offers an avenue for developing highly targeted therapies. By building upon existing research efforts, tailored “neurotransmitter therapies” may provide comprehensive therapeutic benefits for BADs while minimizing adverse impacts on the body’s physiological systems.
